# What we see is what we touch? Sex estimation on the pelvis in virtual anthropology

**DOI:** 10.1007/s00414-023-03034-w

**Published:** 2023-06-19

**Authors:** Sandra Braun, Nicole Schwendener, Fabian Kanz, Sandra Lösch, Marco Milella

**Affiliations:** 1 Department of Physical Anthropology, Institute of Forensic Medicine, University of Bern, Murtenstrasse 24-28, 3008 Bern, Switzerland; 2 Department of Forensic Medicine and Imaging, Institute of Forensic Medicine, University of Bern, Bern, Switzerland; 3 Forensic Anthropology Unit, Center for Forensic Medicine, Medical University of Vienna, Vienna, Austria

**Keywords:** Forensic anthropology, Nonmetric, Metric, DSP2, Computed tomography, Surface scan

## Abstract

**Background:**

Computed tomography (CT) scans are a convenient means to study 3D reconstructions of bones. However, errors associated with the different nature of the observation, e.g. visual and tactile (on dry bone) versus visual only (on a screen) have not been thoroughly investigated.

**Materials and methods:**

We quantified the errors between modalities for sex estimation protocols of nonmetric (categorical and ordinal) and metric data, using 200 dry pelves of archaeological origin and the CT reconstructions of the same bones. In addition, we 3D surface scanned a subsample of 39 pelves to compare observations with dry bone and CT data. We did not focus on the sex estimation accuracy but solely on the consistency of the scoring, hence, the interchangeability of the modalities.

**Results:**

Metric data yielded the most consistent results. Among the nonmetric protocols, ordinal data performed better than categorical data. We applied a slightly modified description for the trait with the highest errors and grouped the traits according to consistency and availability in good, intermediate, and poor.

**Discussion:**

The investigated modalities were interchangeable as long as the trait definition was not arbitrary. Dry bone (gold standard) performed well, and CT and 3D surface scans performed better. We recommend researchers test their affinity for using virtual modalities. Future studies could use our consistency analysis and combine the best traits, validating their accuracy on various modalities.

## Introduction 

The estimation of sex is a crucial parameter during the anthropological analysis of human skeletal remains of forensic or archaeological origin [1–4]. In osteoarchaeological research, sex is a relevant variable when addressing osteobiographies, case studies, and palaeodemographic research questions [1, 5–8]. Conversely, in forensic contexts, biological sex is of primary relevance for individual identification purposes [9]. Although less problematic than other tasks (e.g. estimation of adult age-at-death), the estimation of sex from skeletal remains considers various potential sources of error. This concern is reflected in the currently applied terminology: while in the past, sex was *determined* [10, 11] or *assessed* [12], nowadays the preferred term is the *estimation* of sex [13]. Osteological sex estimation is based on the observation and evaluation of both quantitative (metric) and qualitative (nonmetric) dimorphic traits [13, 14]. Throughout this work, we will refer to these two types of features as metric and nonmetric.

The morphological overlap in sexual dimorphism [1] is accounted for by the ordinal nature of many of the sex estimation methods, e.g. scores 1 to 5, or very gracile to very robust [9], thus accommodating the continuum of sexual dimorphism [1, 9]. Due to its role in reproduction and the correlated selective pressures, the pelvis is the most sexually dimorphic human skeletal structures [1, 5, 6, 12, 15, 16].

Qualitatively, female pelves are characterized by a series of features, including their more outwardly flared geometry, relatively wider inlet, and smaller and more gracile coxal bones [17–20]. These features and the overall lack of substantial differences in pelvic sexual dimorphism across populations make this skeletal structure the primary target for osteological sex estimation [21, 22].

Thus far, various methodological approaches have been published on the quantification of pelvic sexual dimorphism. Despite the increasing availability of molecular techniques, nonmetric and metric protocols are still preferred due to their low economical cost and low invasiveness [3]. Some of the methods most commonly applied by anthropologists, and the focus of the present study, are those of İşcan and Derrick [10], Bruzek [23], Klales et al. [15], and the Diagnose Sexuelle Probabiliste (DSP) method [24, 25]. These methods exemplify nonmetric [10, 15, 23] and metric [24, 25] and accuracy levels have been reported ranging from 86.2 [15] to 100% [26].

### Sex estimation on dry bone and virtual modalities 

Due to ethical concerns, the traditional way of anthropological research on human osteological collections is more and more under scrutiny [27–29]. These osteological collections represent cultural and historical documentation that should be preserved [30, 31]. At the same time, virtual alternatives (e.g. tomographic data, surface scans by means of structured light or laser, photogrammetry) have been established [32–34], and reflective imaging technologies (i.e. surface scans) require dry bones for scanning [35]. This kind of data adds value to the estimation of sex from forensic and clinical radiological images of corpses and patients [36–39] as well as from a bioarchaeological background [8, 40, 41]. In a forensic context, virtual images are nowadays permissible in courtrooms [42–45]. 3D prints of bones are usually preferred over the presentation of macerated dry bones in courtrooms, due to the potential evocation of emotions among the family present [46]. Further fields of application are paleoanthropology and osteoarchaeology [47–52] and evolutionary phylogenetic research [53]. Advantages of the virtual modalities are, for example, the worldwide access to data and consequently the facilitation of long-distance research collaborations [54], the non-invasive investigation of a present-day context [55, 56], and the possibility of advanced shape quantification methods such as geometric morphometrics [57]. However, these approaches are potentially afflicted by at least two issues: (a) the possible presence and extent of deviation between the same observations in the virtual and the physical environment, and (b) the consistency between observations on 3D models obtained by means of different imaging techniques (e.g. CT scans versus 3D surface scans). The quantification of the error affecting analogous versus virtual observations seems especially relevant for nonmetric traits, the scoring of which is typically affected by subjectivity [23, 58–60]. By quantifying the errors associated with each of the sex estimation traits, our understanding of the methods could be impacted. Dissimilarities in the application of a variety of sex estimation traits to different modalities may be owing to the influence of the tactile sensation when handling dry bone versus the visual assessment of 3D models on a screen [36, 58, 60–62].

Previous studies already raised such concerns, while performing tests aimed at quantifying possible discrepancies: Grabherr and colleagues (2009) investigated age and sex estimation on the cranial and the pelvic regions on CT data of 22 individuals. Decker et al. (2011) used CT scans of 100 individuals to compare obstetric measurements and morphological traits on the pelvis. Both studies conclude that CT data are an excellent substitute for dry bone [63, 64], however without directly comparing the modalities and quantifying the associated errors. Another study compared the degree of closure of cranial sutures on ten crania observable on dry bone, 3D models obtained from surface scans, and CT data [61]. In contrast to Grabherr et al. (2009) and Decker et al. (2011), the results indicated differences between the analogous and the 3D surface modalities, while the observations on the CT modality were similar to those on dry bone [61]. The comparison of dry bone and CT images for the nonmetric estimation of age-at-death resulted in recommendations that scanning protocols should be uniform, with a special focus on slice thickness [62]. In another study, the direct comparison of ‘dry’ versus ‘clinical’ CT as well as 3D surface data for the measurement of inter-landmark distances on 14 female and 13 male pelves resulted in the superiority of the ‘dry’ CT data [65]. In contrast, the 3D surface scans of a fractured skull allowed more precise measurements than the multi-detector CT images of the same bone [66]. The authors do, however, add that the statement is limited by the fact that only one specimen was scanned [66]. The DSP method was tested for the interchangeability of the modalities dry bone and CT scans (individual os coxa and pelvic girdle) of 49 pelves [26]. In another study comparing the measurements and geometric variables of the ilium width and the ilium length, as well as the ilium module and the ilium area [67], it was found that the relative technical error of measurement (rTEM) was within the acceptable threshold of 5%, both in the intra- and the interobserver agreement tests. The authors concluded that both modalities could be used with confidence for the tested variables [67]. Although these studies contributed relevant data to discussions about the reliability of virtual observations in anthropological tasks, many of them were based on small sample sizes [61, 62, 64–66].

Since the general trend in anthropology points towards a growing interest in the virtual modalities [52, 62, 64, 68, 69], a comprehensive study on the interchangeability of the analogous and the virtual modalities in sex estimation methods is still lacking [58, 70, 71]. This study attempted to address this research gap and focus on the interchangeability of dry bone and CT on a large sample of pelves (*n* = 200), concentrating on the scoring and measuring consistency, and disregarding the accuracy of the employed methods in estimating sex. Therefore, the use of unidentified archaeological human remains was appropriate for our purpose. In fact, any sample of bones would have been suitable, provided their relative good state of preservation to test as many sex estimation traits as possible. In addition, for a subset of specimens (*n* = 39), we compared observations on digital 3D models obtained by means of 3D surface scans and CT data with observation of the dry bone specimen. We focus on the following points:

- Are analogous pelves and their virtual counterparts interchangeable when trying to estimate sex using nonmetric and metric protocols?
- Is there a difference between observations of dimorphic traits taken on virtual models extracted from CT versus 3D surface scans?
- Which role do the nature of observed variables (nonmetric and metric) and the scoring protocols play on the two former aims?

## Materials and methods

### Materials

Since our institutional forensic database consists of postmortem CT (PMCT) scans taken from fresh bodies, we had no dry bones at our disposal (e.g. [37, 39, 64, 72]). Thus, we selected 200 relatively well-preserved pelves (Table 1) from different archaeological sites and chronologies in Switzerland on which anthropological sex estimation was done following excavation [73–75]. While bone fragmentation cannot be avoided in any anthropological context, we selected the specimens according to a low degree of fragmentation. We chose individuals in order to obtain a balance between the sexes (100 females, 100 males), according to anthropological estimation based on the methods published by Buikstra and Ubelaker [5], Herrmann et al. [76], and White and Folkens [77]. Our intention was to include a wide spectrum of sexual dimorphism; hence, we did not verify sex with proteomic analysis nor DNA analysis (e.g. [78–80). The estimated age-at-death of all individuals ranged between 18 and 80 years. We did not include subadult individuals (< 18 years), nor individuals presenting pathological changes to the pelvis.

**Table 1:**
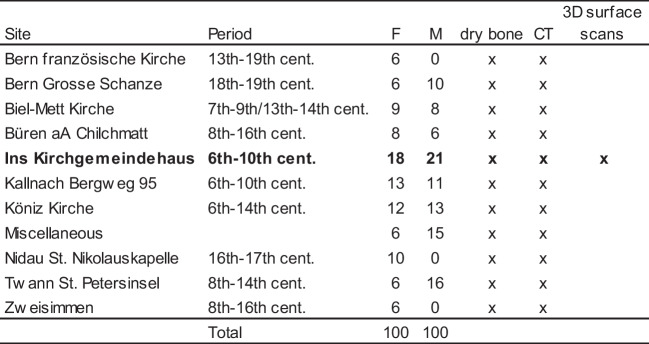
Archaeological sites, chronologies, number of female (F) and male (M) pelves, and modalities (dry bone, CT and 3D surface scans)

Boldface: site used for intra- and interobserver, as well as intermodality agreements on three modalities

### Methods

#### Scoring protocols

We scored the dry bone pelves following the nonmetric sex estimation protocols by İşcan and Derrick [10], Bruzek [23], and Klales et al. [15] and collecting the ten linear DSP2 measurements by means of sliding and spreading calipers [25].

The method by İşcan and Derrick [10] outlines three traits (iliac tuberosity [IT], postauricular space [Pspace] and postauricular sulcus [PS]) to be scored categorically as female (F) or male (M). The postauricular space is regarded as the ‘*most reliable*’ of the three structures [10, 81]. In this study, we used the method due to its simple application for individuals with an intact sacroiliac joint. Bruzek’s method is more complex and contains a variety of conditions for each of the five traits [23]. The composite arch (CA) and ischiopubic proportion (IP) are scored categorically as female (F), indeterminate (I) or male (M). The other three traits (preauricular surface [PSurf], greater sciatic notch [SN] and ischiopubic proportion [IP]) consist of three conditions each (e.g. F, I, M). The final sex estimation per trait is based on the mean score (e.g. female, if at least two conditions are rated female). The ventral arc (VA), the subpubic concavity (SC) and the medial aspect of the ischio-pubic ramus (IR) are comprised in the method that is ordinally scored from 1 (female) to 5 (male) [15]. The publication by Bruzek et al. [25] depicts each variable (acetabulo-symphyseal pubic length [PUM], cotylo-pubic width [SPU], innominate length [DCOX], greater sciatic notch height [IIMT], ischium post-acetabular length [ISMM], iliac breadth [SCOX], spino-sciatic length [SS], spino-auricular length [SA], cotylo-sciatic breadth [SIS], and vertical acetabular diameter [VEAC]). It also includes the linear discriminant analysis formula used for the workable download. The measurements are entered into the database, with the resulting sex estimation given with immediate effect.

All trait scores and linear measurements for each individual and separated per observer, method and modality were entered in an excel sheet. Moreover, we scored and measured the left coxal bone where available and intact. If that was not the case, we observed the traits on the right coxal bone [59, 82].

#### CT and 3D surface scanning

**Fig. 1:**
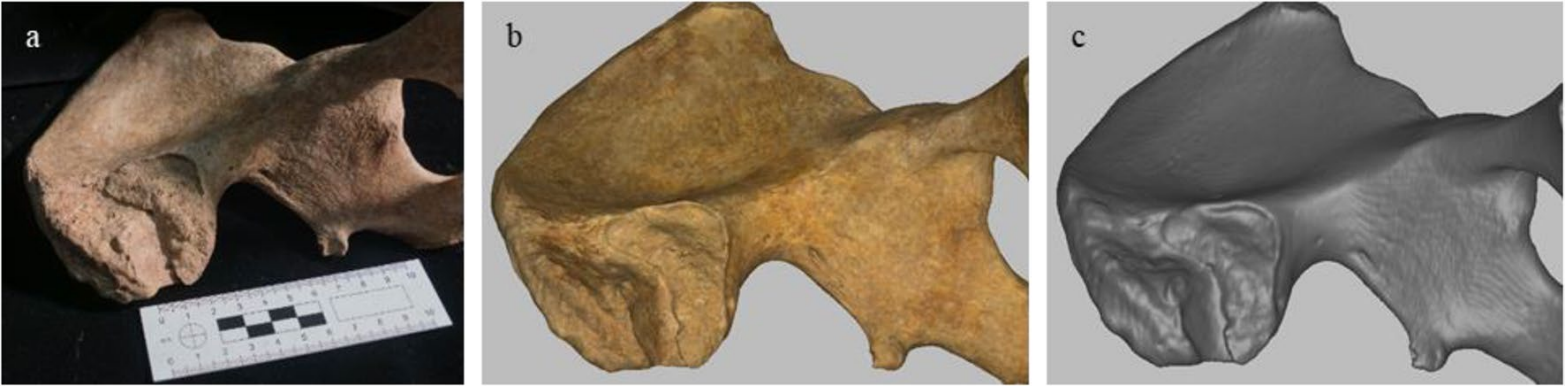
Left coxal bone of Ins Kirchgemeindehaus individual 3543 on **a** dry bone, **b** Artec 3D surface scan, and **c** CT scan

The dry pelves (Fig. 1a) were CT scanned with a Somatom Definition AS 64 (Siemens, Berlin/Munich, Germany). Scanning parameters were 140 kV, 118 to 216 mAs, and a slice thickness of 0.6 mm (increment 0.3 mm). Reconstruction parameters of the matrix of 512 × 512 pixels was a field of view between 200 and 400 mm. We exported all raw tomographic scans as DICOM data from PACS IDS 7 v. 20.2.8.3353 (Sectra, Linköping, Sweden) and reconstructed them in Avizo (Thermo Fisher Scientific Inc., Waltham, MA, USA). We exported the reconstructed bone models again in DICOM format and imported them into Artec Studio software (Artec 3D, Luxembourg). In addition, we obtained 3D surface scans (Fig. 1b) of the 39 pelves (18 female, 21 male) from the Ins Kirchgemeindehaus site (Table 1) with an Artec Spider scanner (Artec 3D, Luxembourg), using the handheld device with the maximum of eight frames per second. We aligned, segmented and reconstructed the 3D surface scans with Artec Studio software, and carried out the subsequent scoring and measuring of both the CT (Fig. 1c) as well as the Artec 3D reconstructions in the Artec Studio software (Artec 3D, Luxembourg). We scanned the pelvic girdles for the assessment of the Pspace trait, and individual coxal bones.

### Data analysis

#### Intra- and interobserver agreement

For the evaluation of the intraobserver agreement, one observer (SB) scored the 39 pelves (female *n* = 18, male *n* = 21) from Ins Kirchgemeindehaus twice on dry bone, on CT as well as on 3D surface scan reconstructions (Table 2). For the interobserver agreement, we compared the first observations of the dry bone, the CT and the 3D surface scan modalities with those of a second observer (MM). We performed the scoring sessions at least two weeks apart from each other. We reported our findings separately for the categorical, ordinal and metric data. The two observers had three and 13 years of experience with imaging techniques, respectively.

**Table 2:**
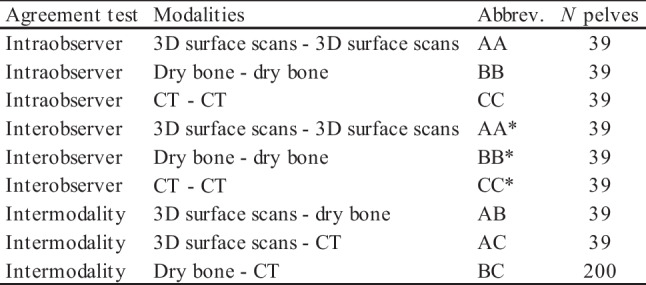
Intra- and interobserver, and intramodality agreement tests

#### Intermodality agreement

We evaluated the intermodality agreement by comparing the deviations in the scores assigned on the same pelvis between the modalities dry bone, CT and 3D surface scans. For the comparisons between the modalities, we used the first scoring sessions of the first observer. We used AB to refer to the comparison between 3D surface scans (Artec) and dry bone (*n* = 39), AC for the comparison between 3D surface scans (Artec) and CT (*n* = 39) and BC for the comparison of dry bone and CT (*n* = 200) (Table 2).

For the categorical data [10, 23], we employed Cohen’s kappa *κ* [83] tests, for the interpretation of which we followed Landis and Koch [84]. The interpretation states that *κ* < 0 indicates a less than chance agreement; *κ* = 0.01 to 0.2 slight agreement; *κ* = 0.21 to 0.4 fair agreement; *κ* = 0.41 to 0.6 moderate agreement, *κ* = 0.61 to 0.8 substantial agreement; and *κ* = 0.81 to 1 almost perfect to perfect agreement [84]. For the ordinally scored method by Klales and colleagues [15], we applied Cohen’s weighted *κ* [83] tests. For all Cohen’s kappa tests, we assumed *κ*-values > 0.6 as acceptable agreement [36, 85]. For the DSP2 method, we analyzed the errors with the rTEM, in %. Although some publications refer to an acceptable rTEM threshold of ≤ 10% [1, 86], we assumed an acceptable rTEM threshold of ≤ 5% [25, 87]. Moreover, we assumed the dry bone modality as the gold standard [36].

#### Trait performance, availability, and scoring consistency

We investigated all traits by depicting the *κ*-value and rTEM ranges across all tests. In addition, we analyzed the DSP2 measurement correlations between the modalities by means of Pearson tests [88].

Osteological remains, especially from a burial context, may be fragmented and badly preserved. Therefore, some sexually dimorphic traits may be unavailable for scoring. We established three scores for the trait availability: score 1 for 80% to 100%, score 2 for availability between 60 and 80%, and score 3 for availability below 60%. Furthermore, consistency scores were either 1 (*κ* > 0.6, rTEM < 5%) or 2 (*κ* < 0.6, rTEM > 5%). Thus, a minimum score of 2, and a maximum of 5 could be reached per trait. We approached the central question of our research, e.g. the interchangeability of modalities by combining trait consistency and availability, resulting in a score between 2 and 5.

We performed all analyses and created all figures in R (version 4.0.4) using the packages *psych* [89], *DescTools* [90], *irr* [91], *fmsb* [92], *ggplot2* [93], and *BlandAltmanLeh* [94].

## Results

### Intra- and interobserver agreements

**Fig. 2:**
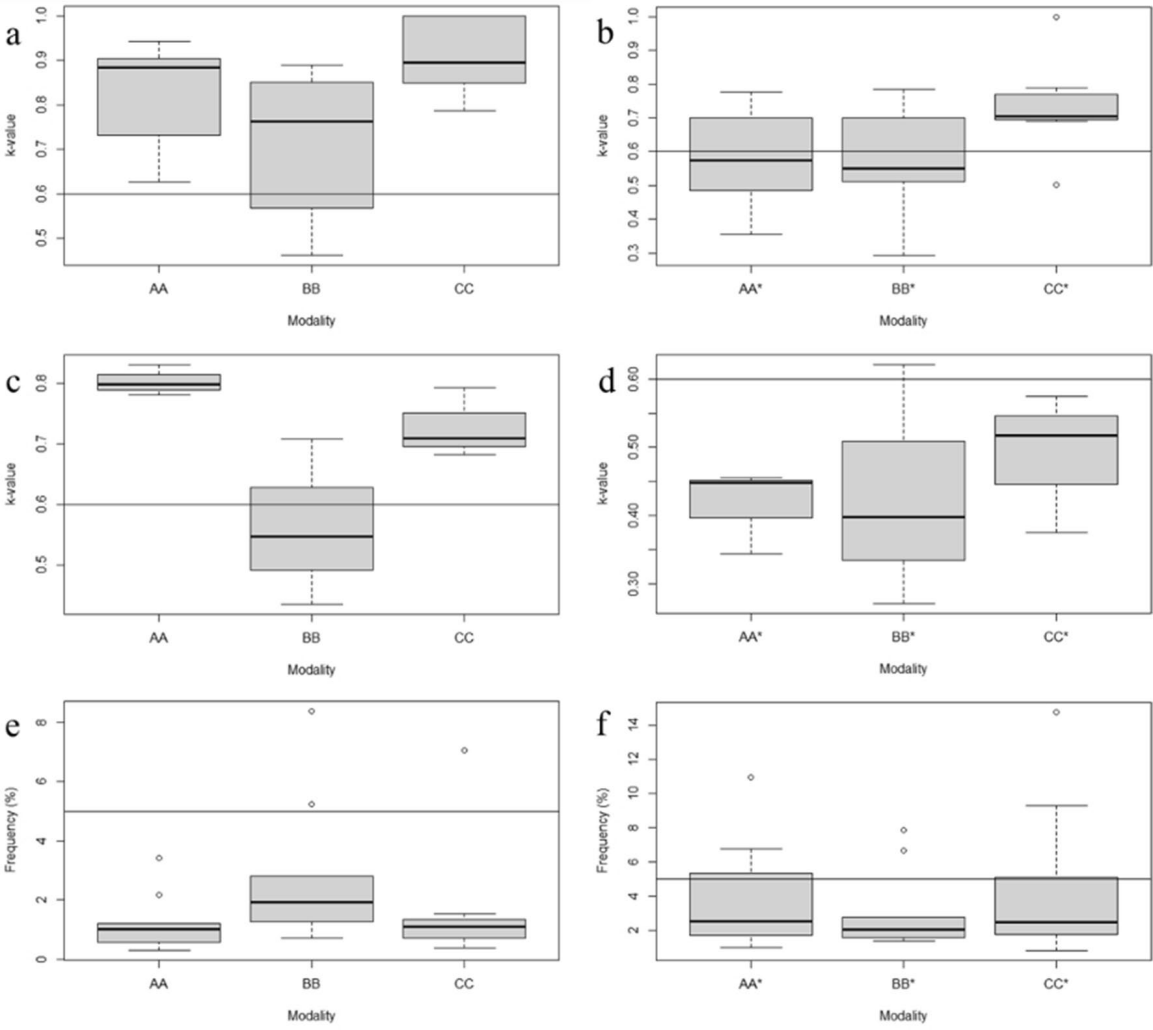
Intra- and interobserver *κ*-value ranges of categorical traits (**a** and **b**), ordinal traits (**c** and **d**) and rTEM of metric traits (**e** and **f**). Horizontal lines indicate the acceptable thresholds (*κ*: 0.6; rTEM: 5%). Intraobserver agreements: AA = surface scan-surface scan; BB = dry bone-dry bone; CC = CT scan-CT scan. Interobserver agreements: AA* = surface scan-surface scan; BB* = dry bone-dry bone; CC* = CT scan-CT scan

For the intraobserver agreement tests, we obtained the highest *κ*-values on the CC modality (mean 0.896, SD = 0.078) for the categorical traits (Fig. 2a). This agreement was closely followed by the AA comparison (mean 0.827, SD = 0.106). The BB comparison yielded a mean of 0.714 (SD = 0.153). In the interobserver agreement test (Fig. 2b), the CC* modality performed best again (mean 0.731, SD = 0.129), followed by the AA* (mean 0.582, SD = 0.135) and the BB* (mean 0.576, SD = 0.147) comparisons, both below the acceptable agreement threshold of 0.6. Cohen’s weighted kappa tests for the ordinal traits (Fig. 2c and d) showed that the AA comparison in the intraobserver agreement test performed best (mean 0.803, SD = 0.021). The agreement of the CC comparison was also acceptable (mean 0.728, SD = 0.047), but the BB comparison and all interobserver tests yielded results below 0.6. The mean rTEM (Fig. 2e and f) were below 5% on all comparisons.

### Intermodality agreements

**Fig. 3:**
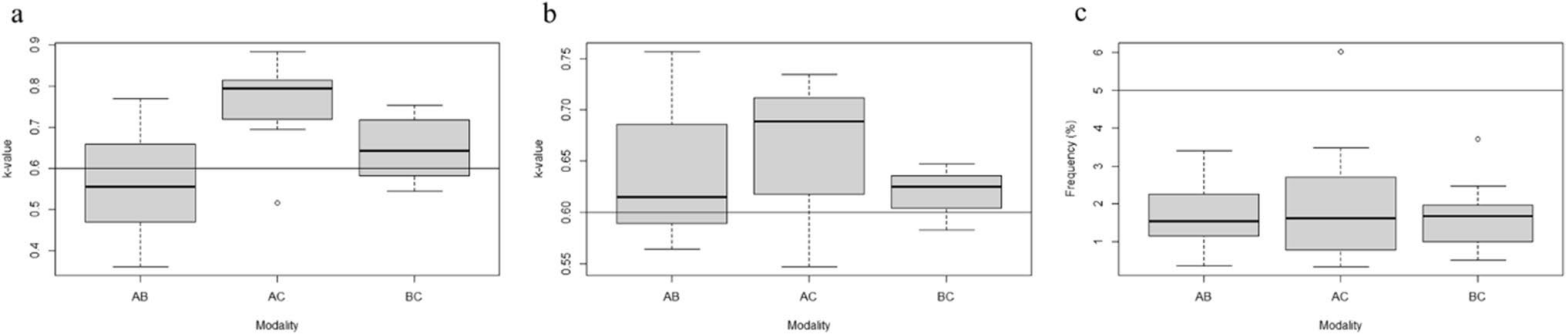
Intermodality agreement *κ*-value ranges of categorical (**a**) and ordinal traits (**b**); rTEM of metric traits (**c**). Horizontal lines indicate the acceptable thresholds (*κ*: 0.6; rTEM: 5%). Intermodality agreements: AB = surface scan-dry bone; AC = surface scan-CT scan; BC = dry bone-CT scan

In the intermodality tests for the ordinal traits (Fig. 3a), we obtained the highest agreement on the AC comparison (mean 0.756, SD = 0.105). The comparison between virtual modalities was followed by the BC comparison (mean 0.647, SD = 0.074), while the AB comparison (mean 0.562, SD = 0.132) was below the acceptable threshold of 0.6. The Cohen’s weighted *κ*-value ranges (Fig. 3b) showed the highest agreement (mean 0.657, SD = 0.080) in the AC comparison. All three comparisons were above 0.6. All intermodality tests for the DSP2 method resulted in rTEM below 5% (Fig. 3c).

### Analysis per trait

**Fig. 4:**
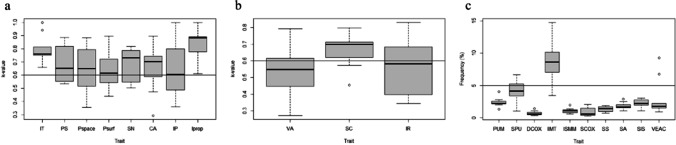
Trait performance for categorical (**a**), ordinal (**b**), and metric traits (**c**). Horizontal lines indicate the acceptable thresholds (*κ*: 0.6; rTEM: 5%). IT = iliac tuberosity; PS = postauricular surface; Pspace = postauricular space; Psurf = preauricular surface; SN = sciatic notch; CA = composite arch; IP = inferior pelvis; Iprop = ischio-pubic proportion; VA = ventral arc; SC = subpubic concavity; IR = medial aspect of the ischio-pubic ramus; PUM = acetabulo-symphyseal pubic length; SPU = cotylo-pubic width; DCOX = innominate length; IIMT = greater sciatic notch height; ISMM = ischium post-acetabular length; SCOX = iliac breadth; SS = spino-sciatic length; SA = spino-auricular length; SIS = cotylo-sciatic breadth; VEAC = vertical acetabular diameter

Regarding the categorical traits, we found the smallest range of *κ*-values in the IT trait, followed by Iprop, which also yielded the highest *κ*-values (mean 0.849, SD = 0.111). We saw the lowest mean *κ*-values in the Psurf and the IP traits (Fig. 4a). For the ordinal traits in Fig. 4b, we obtained the best results (mean 0.665, SD = 0.094) for the SC trait, followed by the IR and the VA trait. We detected a high rTEM for the IIMT measurement when analyzing the performance of the DSP measurements (Fig. 4c).

We display an in-depth evaluation of the *κ*-values and rTEM in Table 3. For the traits IT, Iprop, PUM, DCOX, ISMM, SCOX, SS, SA and SIS, the *κ*-values on all modalities were above 0.6 or the rTEM below 5%, respectively. For the IIMT measurement, only the AA comparison was within the 5% threshold.

**Table 3:**
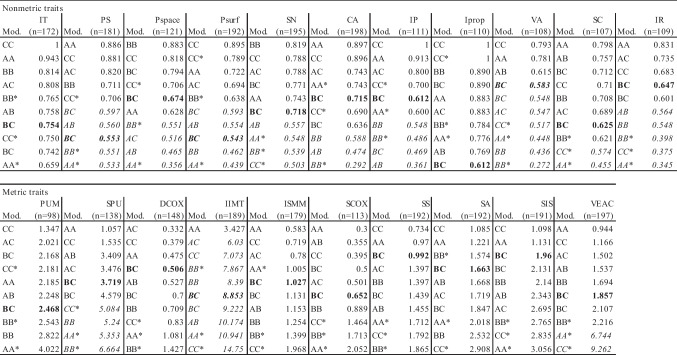
Trait consistency: *κ*-values and rTEM in descending/ascending order per trait; *n* refers to the number of available traits for BC (*n* = 200) comparison, in bold. Italic font indicates *κ*-values < 0.6 and rTEM > 5%

*IT*, iliac tuberosity; *PS*, postauricular surface; *Pspace*, postauricular space; *Psurf*, preauricular surface; *SN*, sciatic notch; *CA*, composite arch; *IP*, inferior pelvis; *Iprop*, ischio-pubic proportion; *VA*, ventral arc; *SC*, subpubic concavity; *IR*, medial aspect of the ischio-pubic ramus; *PUM*, acetabulo-symphyseal pubic length; *SPU*, cotylo-pubic width; *DCOX*, innominate length; *IIMT*, greater sciatic notch height; *ISMM*, ischium post-acetabular length; *SCOX*, iliac breadth; *SS*, spino-sciatic length; *SA*, spino-auricular length; *SIS*, cotylo-sciatic breadth; *VEAC*, vertical acetabular diameter; A, 3D surface scans; B, dry bone; C, CT scans

**Fig. 5:**
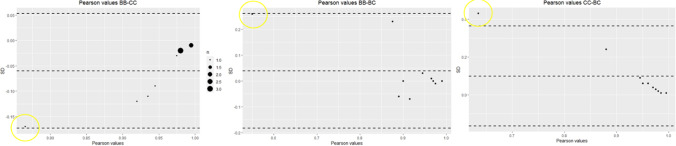
Bland–Altman plots of Pearson correlations of DSP2 measurements of BB-CC, BB-BC and CC-BC (*n* = 200). Yellow circle indicates IIMT measurement

In the Pearson analysis, the IIMT measurement resulted in the lowest mean correlation coefficient *r* (0.65), while the mean of the other nine measurements ranged from 0.92 to 0.99. While all Pearson correlations were significant (*P* < 0.001), the IIMT measurement was on the border or beyond the standard deviation (SD) in the Bland–Altman plots (Fig. 5).

**Fig. 6:**
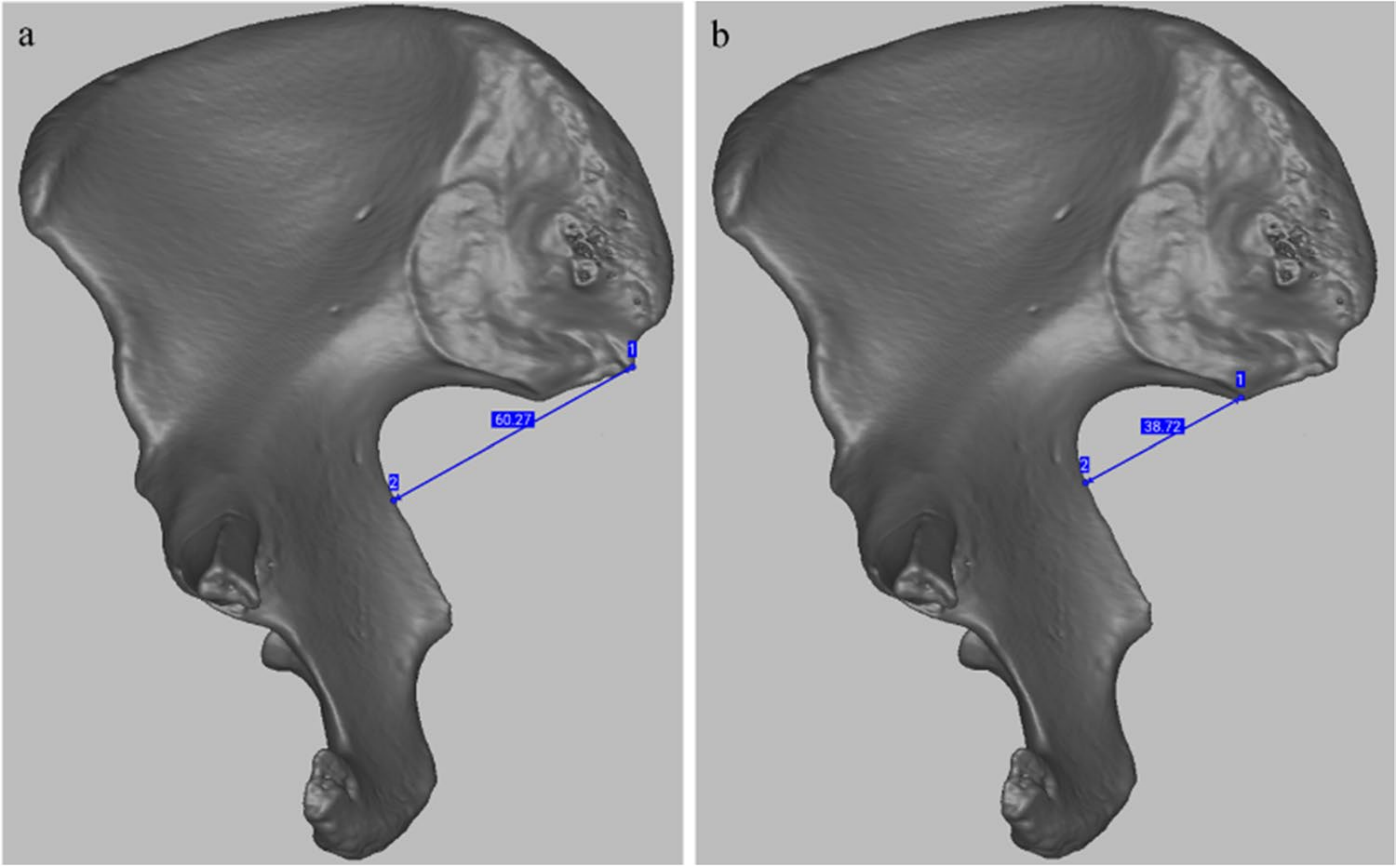
**a** the IIMT measurement from the postero-inferior iliac spine according to White and Folkens (2005), perpendicular to the anterior margin of the sciatic notch. Note the relatively longer distance (60.27 mm versus 38.72 mm); **b** our assumed IIMT measurement; 1 is the intersection between the posterior margin of the sciatic notch and the auricular surface; 2 the point intersecting the anterior margin of the sciatic notch perpendicularly.

**Fig. 7:**
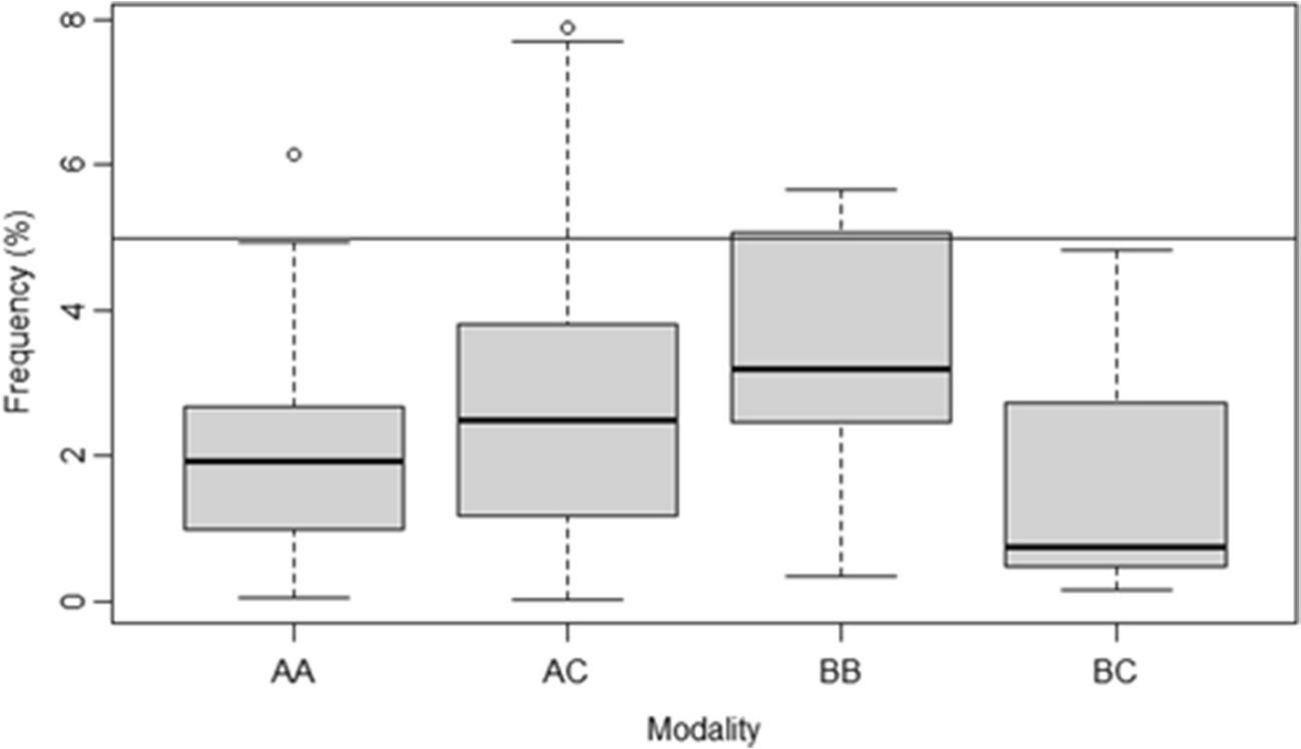
rTEM of IIMT according to our definition. Line indicates rTEM 5%. Agreement tests: AA = surface scan-surface scan; AC = surface scan-CT scan; BB = dry bone-dry bone; BC = dry bone-CT scan

Since the high rTEM of the IIMT measurement could have been due to our interpretation of the location of the postero-inferior iliac spine according to White and Folkens (2005), we tried to focus on the intersection of the auricular surface and the posterior portion of the sciatic notch as a starting point (Fig. 6a and b). This resulted in acceptable rTEM of this measurement (Fig. 7).

### Trait availability

**Fig. 8:**
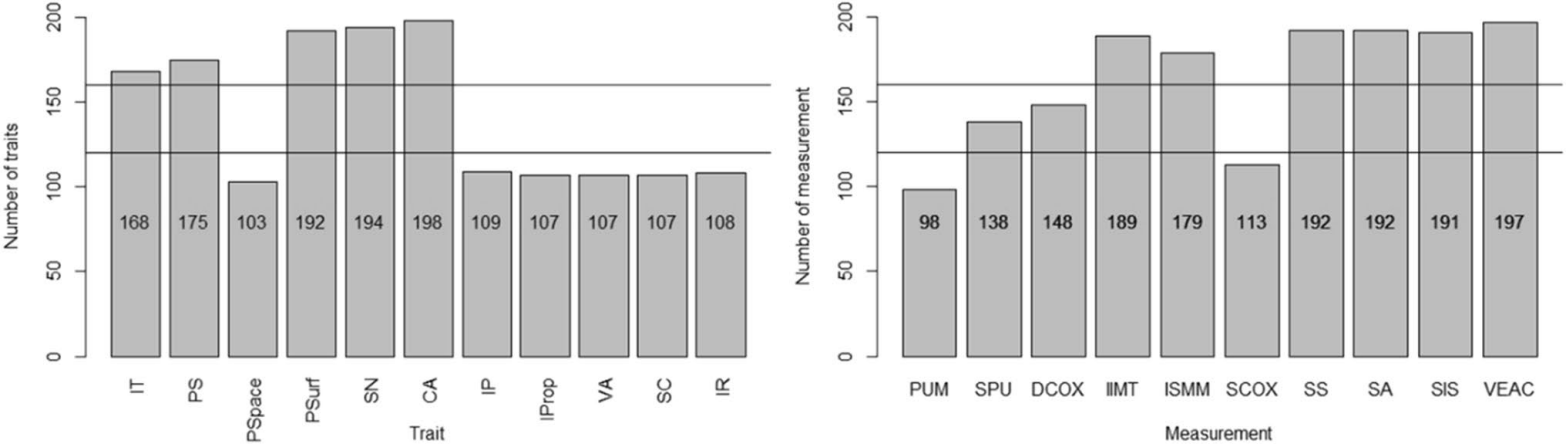
Trait availability: nonmetric (left) and the metric (right) traits, with lines at 60% and 80% availability. Lines indicate 60% and 80% trait availability. IT = iliac tuberosity; PS = postauricular surface; Pspace = postauricular space; Psurf = preauricular surface; SN = sciatic notch; CA = composite arch; IP = inferior pelvis; Iprop = ischio-pubic proportion; VA = ventral arc; SC = subpubic concavity; IR = medial aspect of the ischio-pubic ramus; PUM = acetabulo-symphyseal pubic length; SPU = cotylo-pubic width; DCOX = innominate length; IIMT = greater sciatic notch height; ISMM = ischium post-acetabular length; SCOX = iliac breadth; SS = spino-sciatic length; SA = spino-auricular length; SIS = cotylo-sciatic breadth; VEAC = vertical acetabular diameter

The traits Psurf, SN, IT, PS, CA, IIMT, ISMM, SS, SA, SIS and VEAC were mostly available (> 80%), while SPU and DCOX were available in 60% to 80% of observations. The traits Pspace, IP, Iprop, VA, SC, IR and PUM were available in less than 60% of cases (Fig. 8).

### Consistency analysis

**Fig. 9:**
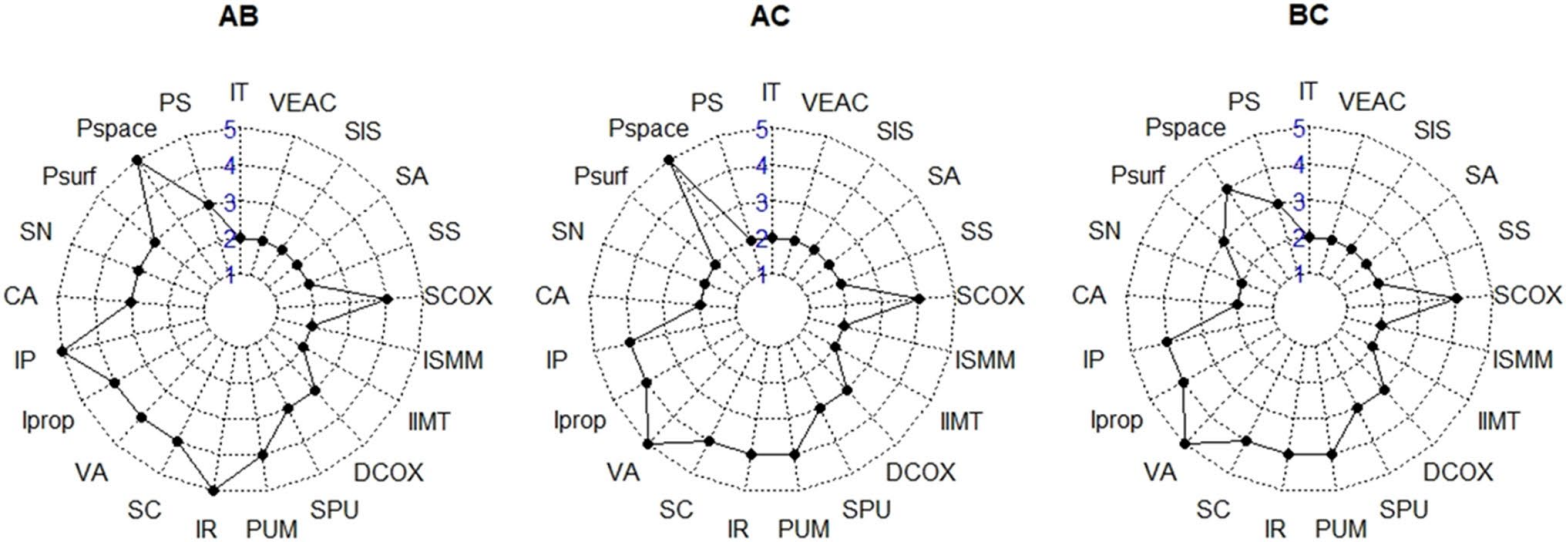
Spiderwebs showing trait consistency and availability in the comparisons between modalities. Scores range from 2 (best) to 5 (poorest) IT = iliac tuberosity; PS = postauricular surface; Pspace = postauricular space; Psurf = preauricular surface; SN = sciatic notch; CA = composite arch; IP = inferior pelvis; Iprop = ischio-pubic proportion; VA = ventral arc; SC = subpubic concavity; IR = medial aspect of the ischio-pubic ramus; PUM = acetabulo-symphyseal pubic length; SPU = cotylo-pubic width; DCOX = innominate length; IIMT = greater sciatic notch height; ISMM = ischium post-acetabular length; SCOX = iliac breadth; SS = spino-sciatic length; SA = spino-auricular length; SIS = cotylo-sciatic breadth; VEAC = vertical acetabular diameter

Based on the above results, we created spiderwebs (Fig. 9), categorizing the trait consistency and availability on the modality pairs AB, AC and BC. The traits IT, IIMT (adapted definition), ISMM, SS, SA, SIS and VEAC obtained the minimal score of 2 in all three comparisons. They are thus the traits with the highest consistency as well as availability. The traits PS, Psurf, SN, CA, Iprop, SC, PUM, SPU, DCOX and SCOX fall into an intermediate class of consistency and availability. Finally, the traits Pspace, IP, VA and IR are poorest in both consistency and availability. Overall, metric traits fared best in terms of consistency, followed by the categorical traits and, finally, by the ordinal traits.

## Discussion

The first research question addressed the degree of agreement between observations of nonmetric and metric sex estimation traits on the pelvis performed on analogous versus virtual models, for which we found satisfactory agreement. The second research question concerned the degree of agreement between virtual models obtained from tomographic data versus 3D surface scans. For this, we found acceptable agreement for all intermodality comparisons. In the following, we discuss our results in detail.

The authors of a previous study [36] analyzed dry pelves and CT scans of the same bones of 14 female and 13 male individuals, and obtained weighted *κ*-values for the intra- and interobserver agreements between 0.61 and 0.9. The *κ*-values of the intermodality agreement tests ranged between 0.58 and 0.84 [36]. Comparing their study to ours, we obtained the highest agreements between modalities, while theirs was within observer. We found intermediate agreement within observer and the lowest agreement between observers, while their study indicated intermediate agreement between observers and the lowest agreement between modalities [36].

To the best of our knowledge, the methods by İşcan and Derrick (1984) and Bruzek (2002) have not been previously tested for consistency on different modalities. Hence, we cannot compare our results to earlier findings, which suggest best agreement within observer, intermediate agreement between modalities and lowest agreement between observers.

Analogous and virtual application of the DSP2 method has been previously compared on 49 pelvic bones [26]. They reported perfect agreement between observers and between modalities, possibly owing to the software used in the study (lhpFusionBox), allowing the ‘virtual palpation’ of anatomical landmarks [26]. Our study does not corroborate the findings of perfect agreement between observers and modalities for the DSP2 method; while our rTEM were acceptable for nine of the ten DSP variables, that was not the case for IIMT. When we adapted the description of this variable slightly for our purposes focusing on the intersection of the auricular surface and the posterior portion of the sciatic notch, we were able to reduce the rTEM below 5% on the different modalities. Thus, irrespective of modality, a precise definition of the traits under analysis is essential. Confounding or imprecise descriptions can lead to inaccuracies in the estimation of sex, and subsequently to a low degree of consistency on any modality [65]. Consequently, we consider the nature of an observation (tactile or visual) less influential on the consistency than the trait description.

Generally, the 3D surface scan and CT (AC) comparison yielded an agreement slightly superior to that of 3D surface scan and dry bone (AB), and dry bone and CT (BC). With reference to the fact that both former modalities are virtual, the greater difference between the analogous and the virtual modalities is self-evident. It seems logical to obtain better agreement between two visual modalities, suggesting a bigger gap between tactile and visual sex estimation than between two visual modalities. Consequently, we summarize that in comparison to dry bone, considered the gold standard, the virtual modalities were even more consistent, thus, interchangeability was superior. This held true for all the different sex estimation protocols used in this study, with better results for the metric than for the nonmetric traits.

In general, and relating to our third research question, a greater error in nonmetric as opposed to metric methods can be ascribed to the inherent subjectivity of qualitative assessments [23, 58, 59]. We were therefore not surprised that the metric traits with a lower degree of subjectivity were more consistent than the nonmetric traits. Moreover, while with categorical traits the scoring options are limited (female or male), ordinal traits are more subtle in the scoring process, allowing the observer a gradual assessment of five scores [36, 59]. These expectations were reflected in our findings, as the source of error was lower with a gradual assessment across the modalities, as opposed to only two categories for selection. However, the categorical data was superior within observer, contrasting with ordinal data.

The challenging fragmentation of human remains from forensic or bioarchaeological contexts due to taphonomic processes [1, 95, 96] is especially pronounced in relation with the pubic bone [97, 98]. To that end, the IP, Iprop, VA, SC, IR and PUM traits were often unavailable for assessment. Other traits with low availability rates (below 60%) were SCOX, owing to eroded margins of the ilium, and Pspace, due to absent or fragmented sacra. Even though we minimized fragmentation in our sample due to a selective sampling process impossible in a forensic context [99], it was still an issue.

For the traits IT, IIMT (adapted definition), ISMM, SA, SS, SIS, and VEAC we found the greatest consistency (interchangeability of modality) as well as availability. The traits Iprop, SN, CA, PS, Psurf, SC, PUM, SPU, DCOX, and SCOX had intermediate qualities of modality interchangeability and availability. The four traits with the poorest consistency and availability in our study were Pspace, IP, VA, and IR. Our explanation for the poor performance of the Pspace trait relates to the fact that the observer needs to articulate the ilium and sacrum. If the two bones are slightly misaligned, results can differ. The trait IP refers to the characterization of the margo inferior ossis coxae, the absence or presence of the phallic ridge and the ischio-pubic ramus aspect [23]. The degree of inferior margin eversion could have been challenging to assess as the orientation on the virtual modalities could easily lead to a misjudgment of the eversion. In the VA trait, it could have been confusing to distinguish a ‘*true ventral arc*’ occurring only in females from a ‘*ridge of bone*’ present in males [100]. The subjectivity of the assessment could have led to the discrepancies in observations on the different modalities. In addition, the absence of tactile sensation on the virtual modalities could have inhibited the precise reproduction of assessing the ridge of bone. The same assessment of a *‘ridge’* in females could have caused the inconsistency of the IR trait [100]. Hence, traits with a description involving a ridge could present a pitfall in the scoring on a non-tactile modality.

To the best of our knowledge, this is the first study to analyze metric and nonmetric methods, the latter including categorical and ordinal data, at the same time encompassing a large dataset of 200 pelves of dry bone and CT data, with a subsample of 3D surface scans. This paper deals with the modality consistency of sex estimation traits. Our study could lay the foundation for future research, focusing on the best and the intermediate group of traits (IT, IIMT [adapted definition], ISMM, SA, SS, SIS, VEAC, Iprop, SN, CA, PS, Psurf, SC, PUM, SPU, DCOX, and SCOX). These traits could be used for a validation study investigating their combined accuracy for estimating sex, thus possibly creating a new set of sex estimation traits [see 62]. The subsequent data would thus encompass not only accuracy but also the trait consistency across modalities.

## Limitations

Limitations to the general applicability of our results include the number of imaging approaches compared, and the specific state of preservation of the skeletal material used in the study. Here, we included two virtual approaches (CT and surface scans) chosen due to their frequent use in anthropological research. However, it would be interesting to test additional approaches (e.g. micro-focus X-ray computed tomography, photogrammetry, laser scanning). The relatively good preservation of the osteological material used in this study likely played a role on our results, especially those from the comparisons between modalities. A useful extension of this study would therefore be the comparison between our results and those obtained by performing the same analyses on highly degraded skeletal remains. Moreover, the state of preservation could be a potential limitation relating to the quality of scans as opposed to a dataset of unfragmented bones as we would need a direct comparison for the depiction of fragments. The participation of an observer without any previous experience with the virtual modalities can be recommended for a future study as levels of observer confidence could vary [101].

As with any sample of bones, the 3D surface scans are challenging on shiny surfaces such as teeth, but also on small foramina and orifices [49].

## Conclusions

Our study confirmed that nonmetric methods are more prone to errors than metric methods, due to the inherent subjectivity of the former. The modalities dry bone and CT are interchangeable within observer, even if on a slightly lower level than the two virtual modalities (CT and 3D surface scans). Irrelevant of their accuracy in estimating sex, IT, IIMT (adapted definition), ISMM, SS, SA, SIS, and VEAC achieved best consistency and availability scores. We conclude that the modalities dry bone, 3D surface scans and CT are interchangeable for the pelvic nonmetric and metric sex estimation techniques applied in this study.

We recommend that future researchers test their affinity for applying a method to the virtual modalities before using it routinely. We support the conclusion of previous work that anthropological curricula should put more weight on developing and improving proficiency in imaging technologies [37, 102]. More pertinent training in this area could encourage the establishment of best practice manuals.

Future research investigating large samples for the consistency of sex estimation techniques on dry bone and virtual modalities will encompass a large sample of skulls. Validating the best and the intermediate traits for sex estimation accuracy could be the objective of another future study, leading to a set of sex estimation traits on the human pelvis and skull that are accurate as well as consistent between the modalities. Moreover, the traits under analysis in this work could be applied to a forensic PMCT dataset of present-day context to test for any possible bias regarding the use of an archaeological sample as in the present research.
